# Selected Metal Concentration in Maternal and Cord Blood

**DOI:** 10.3390/ijerph182312407

**Published:** 2021-11-25

**Authors:** Karolina Kot, Natalia Łanocha-Arendarczyk, Patrycja Kupnicka, Sławomir Szymański, Witold Malinowski, Elżbieta Kalisińska, Dariusz Chlubek, Danuta Kosik-Bogacka

**Affiliations:** 1 Department of Biology and Medical Parasitology, Pomeranian Medical University in Szczecin, Powstańców Wielkopolskich 72, 70-111 Szczecin, Poland; kotkar@pum.edu.pl (K.K.); nlanocha@pum.edu.pl (N.Ł.-A.); ekalist@pum.edu.pl (E.K.); 2 Department of Biochemistry and Medical Chemistry, Pomeranian Medical University in Szczecin, Powstańców Wielkopolskich 72, 70-111 Szczecin, Poland; pkupnicka@gmail.com (P.K.); dchlubek@pum.edu.pl (D.C.); 3 Department of Obstretrics and Pregnancy Pathology, Pomeranian Medical University in Szczecin, Powstańców Wielkopolskich 72, 70-111 Szczecin, Poland; slawomir.szymanski@pum.edu.pl; 4 Department of Obstetrical and Gynecological Nursing, Pomeranian Medical University in Szczecin, Żołnierska 48, 71-210 Szczecin, Poland; witold05@op.pl; 5 Independent Laboratory of Pharmaceutical Botany, Pomeranian Medical University in Szczecin, Powstańców Wielkopolskich 72, 70-111 Szczecin, Poland

**Keywords:** cord blood, metals, maternal blood

## Abstract

Essential and non-essential elements deficiencies may lead to various birth complications. The aim of this paper was to determine calcium (Ca), copper (Cu), iron (Fe), potassium (K), magnesium (Mg), sodium (Na), phosphorus (P), lead (Pb), strontium (Sr), and zinc (Zn) concentrations in maternal blood and cord blood. Whole blood and cord blood samples collected from pregnant women (n = 136) were analyzed for the concentration of the elements by spectrophotometric atomic absorption in inductively coupled argon plasma (ICP-OES). The results showed that Ca, Pb, and Sr concentrations were similar in maternal and cord blood, while Fe and K levels were higher in cord blood than in maternal blood. The cord blood Cu, Na, and Zn concentrations were lower than those in maternal blood, suggesting transplacental transfer of these elements were limited. Moreover, checking the influence of studied elements on the anthropometric parameters of the newborns, we found that the highest number of associations was between Cu in cord blood. Due to the fact that the pregnant women were healthy, and the newborns were without any disorders, we suggest that the values obtained in our study are normal values of studied elements in whole blood and cord blood in patients from Poland.

## 1. Introduction

It has been reported that some xenobiotics, including toxic metals, that have harmful effects on the health of the pregnant woman can also pose a similar or even greater risk to the fetus due to its physiological immaturity [1]. The effect of xenobiotics on the fetus depends on whether it has the ability to penetrate the placental barrier. The placenta has the important function of permeating substances from the maternal system to the fetus [2]. The most important parameters determining transplacental transport are the physicochemical properties of the substance, the physiological state of the placental tissue, and maternal and fetal placental circulation. Blood flow decreases before delivery or as a result of certain conditions such as preeclampsia, hypertension, or diabetes. During uterine contractions, blood flow is reduced, which can result in the retention and accumulation of xenobiotics in the fetus. This is due to the inability of the substance to return through the placenta to the maternal circulation [3]. The mother’s element burden can also be influenced by social determinants, such as socioeconomic status, demographic indicators, as well as lifestyle behaviors such as physical activity, vitamin intake, coffee consumption, and smoking exposure. The diet may affect the toxicokinetics of elements by reducing the absorption and/or toxicity of heavy metals [4].

Many xenobiotics, including heavy metals (e.g., lead), readily penetrate from maternal blood into the blood of the fetus and may cause teratogenic effects [5,6]. However, those effects may also occur in essential element deficiency conditions [7]. For example, calcium deficiency is related to hypertensive disorders and might increase the risk of various problems such as preeclampsia and fetal growth disorders [8]. Hypomagnesemia (magnesium deficiency) during pregnancy is related to a higher health risk for both mother and newborn, including preterm labor, gestational diabetes, restricted fetal growth, intrauterine growth restriction, and preeclampsia [9]. Iron, copper, and zinc deficiencies can cause miscarriage, preterm birth, fetal malformations, or low birth weight [10]. Moreover, deficiencies in essential trace elements may increase the sensitivity and the harmful effects of some toxic elements [11].

The main aims of the study were (1) to determine calcium (Ca), copper (Cu), iron (Fe), potassium (K), magnesium (Mg), sodium (Na), phosphorus (P), lead (Pb), strontium (Sr), and zinc (Zn) concentrations in maternal blood and cord blood; (2) to examine the influence of environmental factors, such as tobacco smoking, dental amalgams filling, vitamin supplementation, and fish and seafood consumption, on the concentration of studied elements in maternal and cord blood; (3) to examine the relationships between elements in the maternal and cord blood; and (4) to examine the influence of studied elements on the anthropometric parameters of the newborns.

## 2. Materials and Methods

### 2.1. Ethics Statement

The use of materials in the study was approved by the Bioethics Committee of the Pomeranian Medical University in Szczecin (KB-0012/76/14 on 13 October 2014). Signed and informed consent was obtained from the patients prior to participation.

### 2.2. Patients Participating in the Study

Pregnant women with healthy, uncomplicated, single pregnancies were recruited between October 2014 and March 2015 from the Neonatal Ward of the Non-Public Health Care Unit at Kutno National Hospital and the Department of Gynecology and Neonatology of the John Paul II Non-Public Health Care Unit in Gryfino. The inclusion criteria were newborns in the absence of perinatal illness. All mothers were healthy, and without risk factors that may determine the size of the newborn such as hypertension, diabetes, infections, etc. Infants were excluded if chromosomal abnormalities and/or congenital malformations were detected during gestation. Blood metals analysis was performed for 136 women and their infants. The patients’ history and questionnaires provided information that is presented in Table 1. Only 24 women (18%) completed the questionnaire.

### 2.3. Determination of Metals in Maternal Blood and Umbilical Cord Blood

The venous maternal blood and the venous umbilical cord blood were collected at the time of delivery. The blood samples were obtained by venipuncture and collected in S-Monovette EDTA (2.6 mL) tubes. The blood samples were transported to the Department of Biology and Medical Parasitology of Pomeranian Medical University in Szczecin within 12 h. Then, whole blood samples (1 mL) were digested in 2 mL of concentrated nitric acid (SuprapurMerck^®^) and 1 mL of 30% hydrogen superoxide (Baker Analyzed). Then, distilled water dilution was added to the solutions of samples to 15 mL for the detection of metals. Determination of Ca, Cu, Fe, K, Mg, Na, P, Pb, Sr, and Zn was performed by spectrophotometric atomic absorption in inductively coupled argon plasma (ICP OES), on a ICAP 7400 (Thermo Scientific). Values below the limit of detection (LoD) were excluded from analysis. The concentrations of elements are expressed as mg/L.

The accuracy of analytical labeling was controlled by determination of the elements in a certified material with a known concentration: Bovine Muscle NIST-SRM 8414. The reference material was prepared in the same manner as samples. The concentration values of the reference materials given by the manufacturers and our determinations are shown in Table 2. Information about QA/QC is given in the Supplementary Materials.

### 2.4. Statistical Analysis

All the data were analyzed with Statistica 10.00 PL software. Shapiro–Wilk analysis was performed to test the normal distribution of the data. Intergroup comparisons were performed using the Mann–Whitney U-test and the Kruskal-Wallis test. The correlations were analyzed on the basis of Spearman’s rank correlation factor (*r*). The significance level was *p* < 0.05.

## 3. Results

The concentration of studied elements in the maternal and cord blood is presented in Table 3. The highest concentration in the maternal blood was of Na, and Pb and Sr had the lowest concentration. In cord blood, K had the highest concentration, and Pb and Sr were at the lowest level. The greatest differences between maternal and cord blood were found in Cu and Zn concentrations, more than twice as high in the maternal blood than in the cord blood. Statistically significant differences between maternal and cord blood were found in all studied elements, apart from Ca and Pb. We did not find any differences in studied element concentration in the maternal blood and cord blood related to the gender of the newborn.

We studied the influence of tobacco smoking, dental amalgams, vitamin supplementation, and fish and seafood consumption on the concentration of studied elements in the maternal and cord blood. However, the aim was not fully achieved because only 18% of women filled out the questionnaires. In statistical analysis, we take into account only patients who answered all questions (n = 24). We found statistically significant higher concentrations of Zn and Fe in the maternal blood of females who confirmed smoking tobacco compared to non-smokers (Zn: 4.23 vs. 2.95 mg/L; Fe: 228.59 vs. 171.08 mg/L). In cord blood, we found only higher Zn level in patients without dental amalgams (1.88 mg/L) than in those who had amalgams filling (1.35 mg/L). No other statistically significant differences were found.

A higher number of revealed correlations was observed in the cord blood than in the maternal blood. In the maternal blood, the strongest correlations (*r* > 0.75) were observed between K and Mg, Ca and Na, Fe and K, and Cu and Ca, while in the cord blood, the strongest relationship was noted between Fe and K, K and Mg, Na and Ca, Fe and Mg, as well as Fe and P. All relationships were synergistic (Table 4 and Table 5).

The interactions between elements in the maternal and cord blood are presented in Table 6. All relationships can be classified as weak or very weak. The strongest correlation was observed between Fe in the maternal blood and Fe in the cord blood (*r* = 0.31). Statistically significant relationships were only synergistic.

We also examined the correlations between the concentrations of Ca, Cu, Fe, K, Mg, Na, P, Pb, Sr, and Zn in the maternal and cord blood and the parameters of the mother, newborn, and placenta (Table 7 and Table 8). A higher number of correlations between the biological parameters of mothers, newborns, and placentas and the concentration of studied elements were found in the cord blood than in maternal blood. All statistically significant relationships can be classified as weak or very weak.

We found no statistically significant interactions in the maternal blood between K, Mg, and Sr concentrations and studied maternal, newborn, and placenta parameters, and between placenta length, placenta width, newborn length, Apgar score in 3 min, and Apgar score in 5 min and studied elements levels. A synergistic relationship in the maternal blood was found only between gestational age and Cu, and also between placenta width and the concentrations of Ca, Na, and Pb. Other correlations found in the maternal blood were antagonistic. The strongest correlation was between the head circumference of newborns and Cu concentration (*r* = −0.29).

In the cord blood, we found no statistically significant interactions between Mg, Pb, and Sr concentrations and the studied parameters, or between maternal age, placenta width, newborn length, chest circumference, and Apgar score in 5 min and the studied element levels. A synergistic relationship in the cord blood was found only between gestational age and Zn concentration, and between placenta width and the concentration of Ca and Na. Other correlations found in the cord blood were antagonistic. The strongest correlation was between head circumference and Zn concentration (*r* = −0.30).

## 4. Discussion

Deviations in the normal metabolism of essential elements may be associated with the occurrence of many disease states [12]. Some trace elements are part of enzymes and others are associated with hormone synthesis. Both excessively low and pathologically high levels of elements in the body can lead to disease [12]. There are also elements, e.g., lead, which are classified as toxic, heavy metals. They have no physiological function in the human body, but the role of these elements in carcinogenesis has been confirmed. The International Agency for Research on Cancer (IARC) classifies Pb in a group of compounds that are likely carcinogenic (group 2A) [13].

Blood is the most common test material used as a biomarker to diagnose deficiency or excess of elements, including toxic metals [14]. Blood reflects current exposure to many elements, but their values very often fluctuate over a wide range of concentrations [15]. Due to the high individual variability, it is often difficult to determine a range of reference concentration values. Since in our study, the pregnancies were normal and the children born were without genetic defects, we suggest that the concentrations obtained in this study are within normal values in women and newborns in Poland. Komarova et al. [16] suggested reference values for trace elements in whole blood. The median values of Cu and Pb obtained in our study were in the reference ranges described by Komarova et al. [16], whereas Zn was two times lower than the optimum level. Mariath et al. [17] also presented a blood reference range and cord reference range. Comparing element blood levels, we found lower levels of Ca, Mg, Na, and Zn and higher levels of Cu, K, and P in our study than in the studies by Mariath et al. [17] and McKeating et al. [18]. In the cord blood, we found a lower level of Na, while K concentration was in the reference ranges described by McKeating et al. [18]. It is important to point out that females in our study were pregnant, and we analyzed the levels of metals by a different method than the aforementioned authors. Moreover, diet and place of residence also affect the levels of elements in the body. The authors [16,18] presented reference values for females living in Australia, and our patients were from Poland.

The concentration of elements in the umbilical cord blood of newborns influences the organism of the developing fetus and the adaptation of the newborn after birth to ectopic life, regulating several vital processes. Some elements are retained by the placental barrier, thus preventing them from entering the developing child’s body; however, the placenta is not an effective barrier for some xenobiotic elements as they are observed in the cord blood of newborns. Such elements include Pb. In our study, Pb concentration in the maternal and cord blood was similar. Furthermore, a weak significant correlation between mother blood and cord blood Pb concentration was noted, which confirms Pb placental transfer. Lead is associated with neurobehavioral and neurological defects, lowered intelligence quotient (IQ), and developmental disorders [19]. Even low blood Pb concentrations can have effects on the ability of children in attention, learning, and productivity at school [20]. The Centers for Disease Control and Prevention (CDC) established the reference blood lead concentration as 5 μg/dL or less [21]. However, there is no safe threshold for blood lead concentrations [22]. In our study, no child had a higher concentration of Pb in cord blood, but two mothers had higher concentrations of Pb in their blood (6 μg/dL and 14 μg/dL). In the study performed in Africa, 75.6% of mothers and 66.8% of newborns had elevated blood lead levels [23]. It is important to point out that blood Pb levels can vary because of changes in hematocrit and Ca levels, plasma volume, and mobilization of Pb from bones during pregnancy [24,25]. In our study, we observed weak correlations in Pb and Ca in maternal blood (*r* = 0.29) and cord blood (*r* = 0.28). Al-Saleh et al. [26] suggested that maternal blood Pb level may be influenced by Ca intake with supplements during pregnancy. In our study, we did not note statistically significant differences in Pb and Ca concentration in maternal blood between patients who were taking supplements during pregnancy and women who were not taking supplementation. Various studies have shown that prenatal Pb exposure was inversely associated with anthropometric parameters of newborns, such as birth weight, birth length, and head circumference [27,28,29]; however, in our study, we did not find any relationship between Pb levels in maternal blood as well as cord blood and anthropometric parameters of newborns.

Strontium is classified as a non-essential heavy metal and its function in the human body is barely known [30]. However, it has been found that Sr is associated with the pathophysiology of preeclampsia [31]. In our study, we did not observe the influence of Sr levels on the parameters of mothers, placentas, and newborns. However, we found weak statistically significant correlation between Sr concentration in maternal and cord blood.

A water–electrolyte disturbance can lead to a number of abnormalities in neonatal metabolism. The consequences can be early and concern the development of the newborn from the fetal period or distant, visible in ectopic life. Zych et al. [32] reported that Na and K levels varied according to a week of gestation. In our study, we did not find any correlations between gestational age and Na or K concentrations in the maternal and cord blood. However, we took blood samples only once, during childbirth. Similar to our study, Czeszyńska et al. [33] also did not find a correlation between Na level and gestational age. Disorders of Na metabolism often occur in newborns with low birth weight, but hypernatremia is much more frequently observed due to high water loss in the first hours of life. Decreased blood Na concentrations are most commonly associated with a syndrome of inappropriate antidiuretic hormone secretion (SIADH), which may occur in meconium aspiration syndrome, pneumonia, or meningitis [34]. Potassium is the most important intracellular cation, and it is essential for maintaining normal muscle contractile function [32].

Recent data suggest that Mg supplementation during pregnancy may prevent some pregnancy complications and improve some health indicators and pregnancy outcomes [35]. It has been shown that Mg may be able to reduce growth restriction of the fetus and preeclampsia and increase birth weight [36,37,38]. Barker et al. [39] found that Mg measured in the umbilical cord correlated significantly with infant birth weight and length. Barbosa et al. [40] also found higher Mg levels in cord blood of newborns with intrauterine growth retardation, as compared with normal-weight newborns. Bermudez et al. [41] found that Mg in maternal blood is inversely and significantly related to all the anthropometric parameters of newborns. In our study, we did not find any associations between Mg and anthropometric parameters of newborns, but it must be pointed out that our newborns had normal birth weight (NBW), and therefore, our results may differ from other researchers who have carried out studies in infants with normal and abnormal parameters.

Essential trace elements, such as Fe, Cu, and Zn, also contribute to the normal development of the newborn during the fetal period. Fe levels were higher in cord blood than in maternal blood. This indicates that the infants receive iron indirectly from their mother’s blood circulation through a prompt and unidirectional passageway. Moreover, similarly to Al-Sahlanee et al. [42], we found a weak but statistically significant correlation between Fe concentration in the maternal blood and cord blood. Srivastava et al. [43] reported that lower availability of Fe in the cord blood may cause a reduction in birth weight and also preterm delivery of a baby. However, in our study, similarly to Bermudez et al. [41], Fe in maternal and umbilical cord blood did not influence birth weight and other anthropometric parameters of newborns.

We found ~60% lower Cu level in the cord blood than in the maternal blood. The result is consistent with previously published papers, which presented a 50–60% decrease in Cu concentration in the fetus [11,44,45,46]. The noted decrease in fetal Cu, a major metallic cofactor in a variety of oxidoreductases, may reduce the potential of cellular oxidative damage in the developing fetus [44]. The appropriate level of Cu is important in the fetus because the liver of a premature infant is immature and cannot accumulate Cu [47]. On the other hand, excess concentration of Cu may be highly toxic to the fetus [48], which is why cord blood Cu (0.31 mg/L in median) seemed to be properly regulated. Kozikowska et al. [49] reported that a decreased level of Cu may result in preterm delivery. We found a positive relationship between Cu concentration in maternal blood and gestational age. Taking into account parameters of newborns, such as head circumference, chest circumference, and Apgar score in the first minute, we found a negative correlation with Cu concentration in the cord blood. Bermudez et al. [41], on the other hand, found that Cu in umbilical cord blood was inversely related to birth weight.

Terrin et al. [50] stated that placental transfer of Zn to the fetus is an active process, mediated by endocytic mechanisms. Zinc deficiency in the fetus is observed only in the presence of severe maternal Zn deficiency because the active transport maintained placental Zn concentration constantly higher than maternal levels. In our study, we found a lower Zn level in the cord blood than in the maternal blood. Similarly, Zhou et al. [51] found lower Zn levels in cord blood clots than in maternal blood clots and concluded that the placenta may play a barrier effect on Zn. However, other studies showed inverse results [52,53]. Bermudez et al. [41] found no associations between anthropometric parameters and Zn level in both umbilical cord and maternal blood. In our study, there was a negative relationship between Zn in maternal blood and the chest circumference of the newborn, and Zn in the cord blood and head circumference of the newborn. Since a high level of Zn may be toxic for the fetus [48], Iwai-Shimada et al. [54] suggested that 0.2 mg/dL of Zn in the cord blood seemed to be optimal. We suggest that our range of Zn concentrations (0.06–0.37 mg/dL) be considered as reference values.

## 5. Conclusions

Some strengths of this paper can be outlined. The first was that it measured 10 elements in maternal and cord blood of a large sample of pregnant women (more than 100 women). We examined the influence of many factors, such as anthropometric parameters of newborns and sociodemographic factors, on the concentration of elements in the maternal and cord blood.

There are some limitations of this study. Only 18% of females filled out the questionnaires. Some statistics were derived from the small number of samples, and it may not be possible to generalize the present findings. Secondly, we measured all the studied elements in whole blood, not in the serum. Therefore, some statistical observations might have been the results of change because there may be a number of potential residual confounding factors, such as hemolysis or interactions between elements.

## Figures and Tables

**Table 1:** General characteristics of the participants (AM, arithmetic mean; SD, standard deviation, Med., median; n, number of participants).

Variables	n	AM ± SD	Med.	Range
Maternal Parameters				
Maternal age	136	28.07 ± 6.15	27.00	16.00–44.00
Gestational age	136	38.99 ± 1.13	39.00	36.00–41.00
Placenta Parameters				
Weight of the placenta (g)	136	516.69 ± 139.03	500.00	100.00–950.00
Length of the placenta (cm)	136	17.83 ± 3.82	17.00	10.00–28.00
Width of the placenta (cm)	136	17.90 ± 3.41	18.00	10.00–28.00
Newborn Parameters				
Weight of the newborn (g)	136	3356.47 ± 357.67	3360.00	2540.00–4000.00
Height of the newborn (cm)	136	53.98 ± 2.54	54.00	48.00–61.00
Head circumference (cm)	67	34.31 ± 1.46	35.00	31.00–38.00
Chest circumference (cm)	67	33.64 ± 1.39	34.00	31.00–36.00
Apgar score in 1 min	136	9.28 ± 0.89	10.00	7.00–10.00
Apgar score in 3 min	136	9.67 ± 0.59	10.00	8.00–10.00
Apgar score in 5 min	136	9.63 ± 0.58	10.00	8.00–10.00
**Variables**		**Category**		**Count**
Newborn’s gender		Female/Male		64/72
Smoking		Yes/No		15/9
Dental amalgam		Yes/No		8/16
Vitamin supplementation		Yes/No		9/15
Fish consumption		No		2
		Once a month		5
		Several times a month		10
		Once a week		6
		Several times a week		1
Seafood consumption		No		12
		Once a month		10
		Several times a month		0
		Once a week		1
		Several times a week		1

**Table 2:** Analysis of reference material Bovine Muscle NIST-SRM 8414.

Element	Reference Values (mg/L)	Percentage of Reference Values
Ca	145 ± 20	95.9
Cu	2.84 ± 0.45	106.0
Fe	71.2 ± 9.2	108.4
K	15,170 ± 370	100.9
Mg	960 ± 95	102.7
Na	2100 ± 80	103.5
P	8360 ± 450	104.3
Pb	0.38 ± 0.24	128.9
Sr	0.052 ± 0.015	113.5
Zn	142 ± 14	95.1

**Table 3:** The concentration of Ca, Cu, Fe, K, Mg, Na, P, Pb, Sr, and Zn in the maternal and cord blood. The concentration is expressed in mg/L (AM, arithmetic mean; SD, standard deviation; Med., median).

	Maternal Blood			Cord Blood			*p*
	AM ± SD	Med.	Range	AM ± SD	Med.	Range	
**Essential elements**							
Ca	32.35 ± 8.70	31.65	9.66–67.53	31.39 ± 8.95	30.24	14.61–83.93	0.19
Cu	0.91 ± 0.41	0.89	0.23–3.99	0.34 ± 0.13	0.31	0.12–1.06	<0.001
Fe	187.09 ± 65.78	185.55	35.18–444.09	263.09 ± 85.41	254.31	66.15–493.53	<0.001
K	819.95 ± 235.89	788.24	341.56–1658.04	1118.21 ± 326.08	1056.52	388.94–2095.78	<0.001
Mg	15.89 ± 4.64	15.42	5.82–33.65	16.99 ± 4.53	16.24	8.05–32.55	<0.05
Na	1035.87 ± 261.60	1010.47	366.99–1965.01	858.92 ± 228.30	814.99	443.30–1915.04	<0.001
P	201.21 ± 117.39	178.21	66.21–979.79	237.10 ± 159.78	206.49	101.31–1340.17	<0.001
Zn	2.94 ± 1.10	2.89	0.81–7.88	1.37 ± 0.57	1.26	0.58–3.65	<0.001
**Non-essential elements**							
Pb	0.02 ± 0.01	0.01	<0.00–0.14	0.02 ± 0.01	0.02	<0.00–0.05	0.12
Sr	0.02 ± 0.01	0.02	0.01–0.06	0.02 ± 0.01	0.02	0.01–0.06	<0.01

**Table 4:** Spearman’s rank correlation cofactors between elements in the maternal blood.

	Ca	Cu	Fe	K	Mg	Na	P	Pb	Sr	Zn
Ca	1.00									
Cu	**0.76**	1.00								
Fe	**0.27**	**0.30**	1.00							
K	**0.54**	**0.47**	**0.81**	1.00						
Mg	**0.69**	**0.56**	**0.73**	**0.88**	1.00					
Na	**0.82**	**0.66**	**0.43**	**0.64**	**0.74**	1.00				
P	**0.44**	**0.46**	**0.69**	**0.69**	**0.71**	**0.49**	1.00			
Pb	**0.29**	**0.22**	**0.30**	**0.40**	**0.41**	**0.33**	**0.25**	1.00		
Sr	**0.49**	**0.31**	0.14	**0.31**	**0.35**	**0.42**	0.09	**0.34**	1.00	
Zn	**0.45**	**0.50**	**0.80**	**0.71**	**0.74**	**0.52**	**0.69**	**0.43**	0.16	1.00

Note: bolded is statistically significant, *p* < 0.05, the same as following table.

**Table 5:** Spearman’s rank correlation cofactors between elements in the cord blood.

	Ca	Cu	Fe	K	Mg	Na	P	Pb	Sr	Zn
Ca	1.00									
Cu	**0.52**	1.00								
Fe	**0.40**	**0.72**	1.00							
K	**0.57**	**0.66**	**0.90**	1.00						
Mg	**0.67**	**0.72**	**0.80**	**0.87**	1.00					
Na	**0.86**	**0.50**	**0.42**	**0.57**	**0.63**	1.00				
P	**0.39**	**0.65**	**0.76**	**0.73**	**0.70**	**0.31**	1.00			
Pb	**0.28**	**0.19**	**0.28**	**0.27**	**0.37**	**0.24**	**0.18**	1.00		
Sr	**0.43**	**0.27**	**0.23**	**0.31**	**0.38**	**0.38**	0.12	**0.30**	1.00	
Zn	**0.55**	**0.74**	**0.62**	**0.61**	**0.65**	**0.45**	**0.56**	**0.22**	**0.32**	1.00

**Table 6:** Spearman’s rank correlation cofactors between elements in the maternal and cord blood.

		Maternal Blood									
		Ca	Cu	Fe	K	Mg	Na	P	Pb	Sr	Zn
Cord Blood	Ca	**0.18**	0.08	0.06	0.14	0.16	0.12	0.09	0.04	0.05	0.03
	Cu	0.04	0.02	**0.19**	0.10	0.13	0.05	0.11	0.07	0.01	0.10
	Fe	**0.18**	0.17	**0.31**	**0.26**	**0.29**	**0.24**	**0.22**	0.12	0.17	**0.23**
	K	**0.23**	0.15	**0.25**	**0.27**	**0.28**	**0.26**	**0.22**	0.11	0.16	**0.18**
	Mg	**0.18**	0.08	**0.21**	**0.22**	**0.27**	**0.20**	**0.18**	0.18	0.13	0.16
	Na	0.10	0.05	0.01	0.09	0.13	0.09	0.02	0.05	0.07	−0.02
	P	0.15	0.08	0.14	0.16	0.15	0.12	0.10	0.07	0.10	0.06
	Pb	0.11	0.05	0.02	0.07	0.11	0.13	0.00	**0.26**	0.08	0.05
	Sr	0.16	0.10	0.15	**0.17**	**0.20**	**0.21**	**0.18**	0.05	**0.23**	0.06
	Zn	0.13	0.09	0.11	0.09	0.10	0.09	0.08	0.08	0.11	0.06

**Table 7:** Spearman’s rank correlation cofactors between biological parameters of the mother, newborn, and placenta, and the concentrations of Ca, Cu, Fe, K, Mg, Na, P, Pb, Sr, and Zn in the maternal blood (bolded is statistically significant, *p* < 0.05).

	Ca	Cu	Fe	K	Mg	Na	P	Pb	Sr	Zn
Gestational age	0.04	**0.18**	0.10	0.06	0.04	0.03	0.15	0.05	−0.01	0.15
Maternal age	−0.01	−0.08	−0.07	0.02	−0.03	−0.06	−0.08	−0.02	0.10	−0.13
Placenta weight	−0.03	−0.12	−0.11	−0.03	−0.04	0.00	−0.06	−0.01	−0.05	−0.16
Placenta length	−0.05	−0.10	−0.04	−0.04	−0.02	−0.03	0.10	−0.02	−0.04	−0.03
Placenta width	**0.18**	0.05	0.09	0.07	0.16	**0.18**	0.11	**0.18**	0.14	0.16
Newborn weight	−0.04	−0.14	−0.08	−0.02	−0.06	−0.07	−0.01	−0.02	−0.05	−0.12
Newborn length	0.07	−0.01	0.05	0.13	0.04	0.07	0.14	0.15	0.04	0.10
Head circumference	−0.04	**−0.29**	−0.17	−0.05	−0.03	0.06	−0.21	0.04	0.08	−0.23
Chest circumference	0.02	−0.17	−0.25	−0.13	−0.09	−0.05	**−0.25**	0.10	0.11	**−0.25**
Apgar score in 1 min	**−0.23**	−0.15	−0.10	−0.15	−0.15	−0.19	−0.07	−0.04	−0.11	−0.13
Apgar score in 3 min	0.19	0.17	−0.10	0.03	0.08	0.08	−0.03	0.06	0.04	−0.01
Apgar score in 5 min	0.10	0.13	0.07	0.14	0.23	0.26	0.16	0.01	0.13	0.08

**Table 8:** Spearman’s rank correlation cofactors between biological parameters of the mother, newborn, and placenta, and the concentrations of Ca, Cu, Fe, K, Mg, Na, P, Pb, Sr, and Zn in the cord blood (bolded is statistically significant, *p* < 0.05).

	Ca	Cu	Fe	K	Mg	Na	P	Pb	Sr	Zn
Gestational age	0.08	0.14	0.08	0.08	0.06	0.01	0.12	−0.04	−0.03	**0.29**
Maternal age	−0.02	−0.12	−0.01	0.01	−0.04	−0.03	0.00	0.15	0.10	−0.02
Placenta weight	0.05	**−0.20**	**−0.19**	−0.15	−0.09	0.11	**−0.19**	0.05	−0.03	**−0.21**
Placenta length	0.07	−0.12	−0.04	−0.04	−0.03	0.09	0.11	−0.03	−0.04	−0.06
Placenta width	**0.24**	0.04	0.02	0.09	0.10	**0.20**	0.06	0.09	0.16	0.14
Newborn weight	0.02	−0.17	−0.14	−0.09	−0.11	0.05	−0.09	−0.01	0.00	−0.13
Newborn length	0.11	−0.01	0.00	0.07	0.01	0.12	0.07	0.11	−0.01	0.04
Head circumference	0.04	**−0.29**	−0.16	0.03	−0.03	0.09	−0.17	−0.01	0.10	**−0.30**
Chest circumference	0.10	**−0.26**	−0.16	−0.09	−0.06	0.15	−0.19	0.08	0.09	−0.19
Apgar score in 1 min	−0.04	**−0.19**	−0.15	**−0.18**	−0.12	0.02	−0.11	−0.09	−0.08	−0.14
Apgar score in 3 min	0.05	−0.06	0.02	0.02	0.07	−0.01	0.05	0.19	0.02	0.04
Apgar score in 5 min	−0.01	−0.18	0.04	0.16	0.16	0.02	0.02	0.19	0.15	−0.21

## Data Availability

The data presented in this study are available on request from the corresponding author.

## Supplementary Materials

The following are available online at https://www.mdpi.com/article/10.3390/ijerph182312407/s1, Table S1: Instrumental operating parameters for ICP OES, ICAP 7400, Thermo Scientific, Table S2: Instrumental detection limits (LoD) and Limit of quantitation (LoQ) for ICAP OES on a ICAP 7400, Thermo Scientific, Table S3: Mean concentration (C1-C3) and relative standard deviation (RDS; %) of the standard sample (1.000 mg/L) measured in 3 days of analysis, Table S4: Table showing reference values with SD of SRM 8414 given by NIST and measured values of the same SRM in four quarters (q1-q4) of a year. Bias and RPD (Relative Percent Difference) were calculated according to the description in the text. RPD I represents the difference between q1 and q2, RPD II–q2 and q3, RPD III–q3 and q4, Table S5: Internal standard–yttrium (Y) recovery range of the standard and samples, Figure S1: A standard curve and limit detection (LoD) for Ca, Cu, Fe, K, Mg, Na, P, Pb, Sr, and Zn by inductively couples plasma atomic emission spectroscopy (ICP OES) on ICAP 7400, Thermo Scientific.
