# Supplementary material for: Selected Metal Concentration in Maternal and Cord Blood
Source: Int J Environ Res Public Health. 2021 Nov 25;18(23):12407. doi: 10.3390/ijerph182312407 (PMC8656657; doi:10.3390/ijerph182312407)
Supplement: Supplementary file 1 [file ijerph-18-12407-s001.zip › ijerph-1421072-supplementary.pdf]

Supplementary Materials

1. Information about QA/QC data

Concentrations of studied elements in final solutions were digested with ICP OES, ICAP 7400 Thermo Scientific. The operating condition of the ICP device is presented in **Table S1**.

Table S1. Instrumental operating parameters for ICP OES, ICAP 7400, Thermo Scientific

Parameter (unit)	Value
RF power (W)	1150
Plasma flow (L/min)	12
Auxiliary flow (L/min)	0.5
Nebulizer model	K-Type Concentric
Nebulizer gas flow (L/min)	0.5
Sample flow (mL/min)	0.8
Gas	Argon
Plasma viewing	Duo
Read delay per replicate (s)	25
Number of replicates	3

Quality assurance and control (QA/QC) procedures were carried out for estimation of the studied metals. All glassware used for analysis were acidwashed and thoroughly rinsed with deionized water. The methods of trace element measurement were validates and controlled by preparation and analyses of standard solutions, calibration of the instrument, daily runs of method blanks, duplicated and replicates and certified materials with each analytical cycle.

A blank sample was analyzed after every batch (15 measurements). Each sample was measured three times, and the relative standard deviation (RSD) was calculated by the software (Qtegra™, Thermo Scientific). A standard curve was plotted before each analysis. The analysis of the sample was performed only if the correlation linearity of the standard curve (R^2) was greater than 0.999. The wavelength used for each element, R^2, limits of detection (LoD) and limits of quantitation (LoQ) for each analyzed element are given in **Table S2 and Figure S1**.

Values below limit of detection (LoD) were excluded from statistical analysis. 23 samples (8.5%) had Pb concentration below LoD.

Table S2. Instrumental detection limits (LoD) and Limit of Quantitation (LoQ) for ICP AES on a ICAP 7400, Thermo Scientific

Element	Wave Length [nm]	R^2	LoD [mg/L]	LoQ
Ca	396.847	0.9998	0.0043	0.0130
Cu	224.700	1.0000	0.0006	0.0018
Fe	238.204	1.0000	0.0004	0.0012
K	766.490	0.9999	0.0061	0.0185
Mg	280.270	1.0000	0.0004	0.0012
Na	589.592	0.9990	0.0355	0.1076
P	178.284	1.0000	0.0147	0.0445
Pb	220.353	0.9999	0.0028	0.0085

Sr	421.552	1.0000	0.0002	0.0006
Zn	206.200	1.0000	0.0001	0.0003

To ensure the proper quality of the measurements, in each analysis, the concentration of the standard sample (1.000 mg/L) was measured (**Table S3**), and the one-year evaluation of reference material (SRM 8414, NIST) was performed (**Table S4**).

Further calculation of standard deviation (SD), coefficient of variation (CV) - the SD expressed as a percent of the mean (CV = standard deviation/mean x 100), method bias, and relative percent difference (RPD) were made to ensure the precision of the method.

RPD % was calculated according to the formula below, where qx and qy correspond to successive values obtained during the analysis of the reference material during one calendar year (q1-q4).

$$RPD\ \% = |[qx-qy]| \ / \ [[qx+qy]/2] \times 100$$

The calculation of the method bias was performed by finding the difference between the values for each elements in SRM 8141 given by manufacturer and the obtained values (q1-q4). The errors in each quarter of a year were added up and divided by the number of measurements. All of the measured values were within the acceptable range specified by the manufacturer of the SRM 8414.

Table S3. Mean concentration (C1-C3) and relative standard deviation (RDS; %) of the standard sample (1.000 mg/L) measured in 3 days of analysis.

Element	C1 (mg/L)	C2 (mg/L)	C3 (mg/L)	RSD 1 (%)	RSD 2 (%)	RSD 3 (%)
**Ca**	1.049	1.074	1.054	0.6	1.0	0.8
**Cu**	1.049	1.095	1.073	0.8	0.7	0.2
**Fe**	1.051	1.043	1.070	0.2	0.6	0.3
**K**	0.962	1.003	1.011	2.0	0.7	2.2
**Mg**	1.053	0.972	0.964	0.4	0.8	0.4
**Na**	1.142	1.098	1.150	0.4	1.5	1.2
**P**	1.015	1.173	1.022	0.4	0.1	0.1
**Pb**	1.046	1.123	1.103	0.3	0.8	0.4
**Sr**	1.016	0.979	1.004	0.3	0.5	0.5
**Zn**	1.017	1.071	1.068	0.3	0.8	0.1

Table S4. Table showing reference values with SD of SRM 8414 given by NIST and measured values of the same SRM in four quarters (q1-q4) of a year. Bias and RPD (Relative Percent Difference) were calculated according to the description in the text. RPD I represents the difference between q1 and q2, RPD II – q2 and q3; RPD III – q3 and q4.

Element	Reference values [mg/L]	CV %	Measured Values q I	q II	q III	q IV	SD	CV %	Bias	RPD % I	RPD% II	RPD % III	RPD % Mean
**Ca**	145±20	13.79	152.00	141.00	138.00	139.00	5.59	3.92	2.50	**7.51**	**2.15**	**0.72**	**3.46**
**Cu**	2.84±0.45	15.85	3.12	3.06	3.20	3.01	0.07	2.29	-0.26	**1.94**	**4.47**	**6.12**	**4.18**
**Fe**	71.2±9.2	12.92	75.80	76.10	78.30	77.20	0.99	1.28	-5.65	**0.39**	**2.85**	**1.41**	**1.55**
**K**	15170±370	2.44	14991.00	15290.00	15590.00	15313.00	212.01	1.39	-126.00	**1.97**	**1.94**	**1.79**	**1.90**
**Mg**	960±95	9.90	923.00	923.00	912.00	986.00	29.21	3.12	24.00	**0.00**	**1.20**	**7.80**	**3.00**
**Na**	2100±80	3.81	2169.00	2146.00	2156.00	2174.00	10.99	0.51	-61.25	**1.07**	**0.46**	**0.83**	**0.79**
**P**	8360±450	5.38	8674.00	8874.00	8753.00	8716.00	74.57	0.85	-394.25	**2.28**	**1.37**	**0.42**	**1.36**
**Pb**	0.38±0.24	63.16	0.50	0.48	0.45	0.49	0.02	3.90	-0.10	**4.08**	**6.45**	**8.51**	**6.35**
**Sr**	0.052±0.015	28.85	0.06	0.04	0.05	0.06	0.01	11.59	0.00	**25.74**	**10.75**	**18.52**	**18.34**
**Zn**	142±14	9.86	148.00	138.00	149.00	135.00	6.10	4.28	-0.50	**6.99**	**-7.67**	**9.86**	**8.17**

Also, the analysis of Standard Reference Material (NIST RM 8414) was performed each day of whole blood analysis. The results of the analysis are included in the main text (Table 2). The recovery of studied elements was in the range of accepted values presented in the manufacturer's certificate.

All measurements were corrected by the software (Qtegra™, Thermo Scientific) for variations in the sample delivery system and the loss of analyte during sample preparation based on the internal standard (Y) recovery (**Table S5**). The software automaticaly eliminates the measurements that show less than 80% or more than 120% yttrium recovery. The analysis of samples outside the acceptable range was repeated.

Table S5. Internal standard – yttrium (Y) recovery range of the standards and samples.

Element	Y recovery range [%]
Standards	92-98.7
Samples	89.9-103.2

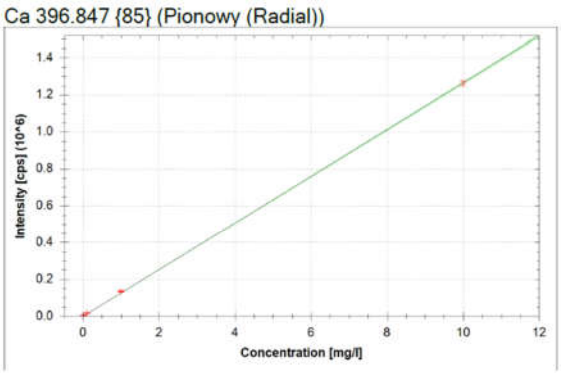

f(x) = 126497.1661*x
R^2 = 0.9998
BEC = 0.000 mg/l
LoD = 0.0043 mg/l

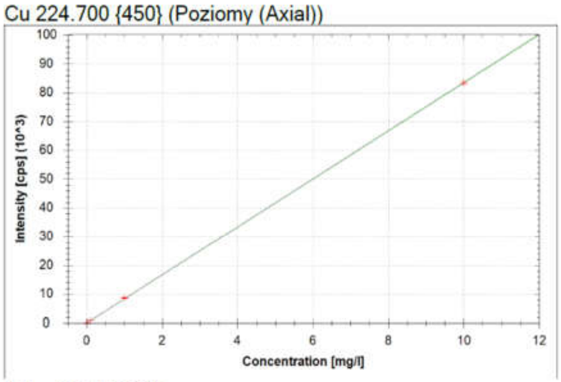

f(x) = 8354.2633*x
R^2 = 1.0000
BEC = 0.000 mg/l
LoD = 0.0006 mg/l

Fe 238.204 {142} (Poziomy (Axial))

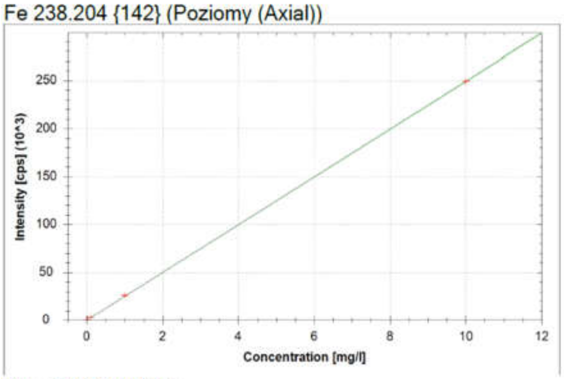

f(x) = 24924.6874*x
R^2 = 1.0000
BEC = 0.000 mg/l
LoD = 0.0004 mg/l

K 766.490 {44} (Pionowy (Radial))

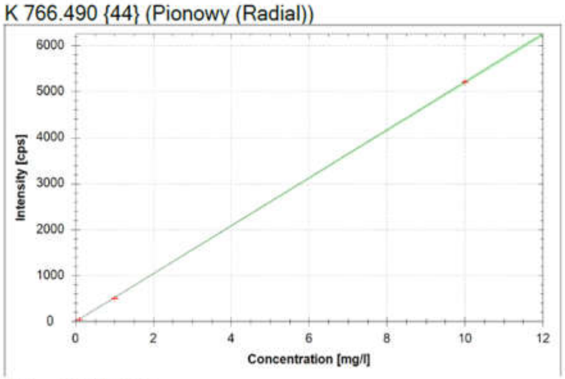

f(x) = 520.8541*x
R^2 = 0.9999
BEC = 0.000 mg/l
LoD = 0.0061 mg/l

Mg 280.270 {120} (Pionowy (Radial))

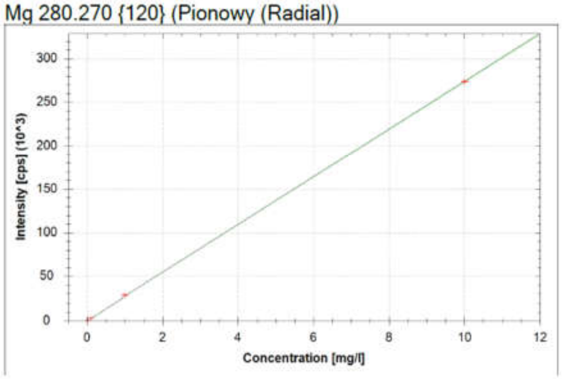

f(x) = 27392.9340*x
R^2 = 1.0000
BEC = 0.000 mg/l
LoD = 0.0004 mg/l

Na 589.592 {57} (Pionowy (Radial))

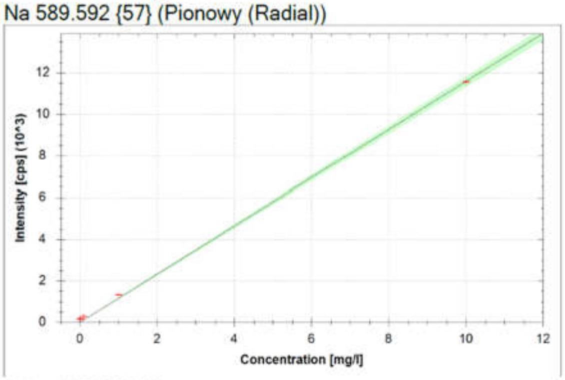

f(x) = 1158.7249*x
R^2 = 0.9990
BEC = 0.000 mg/l
LoD = 0.0355 mg/l

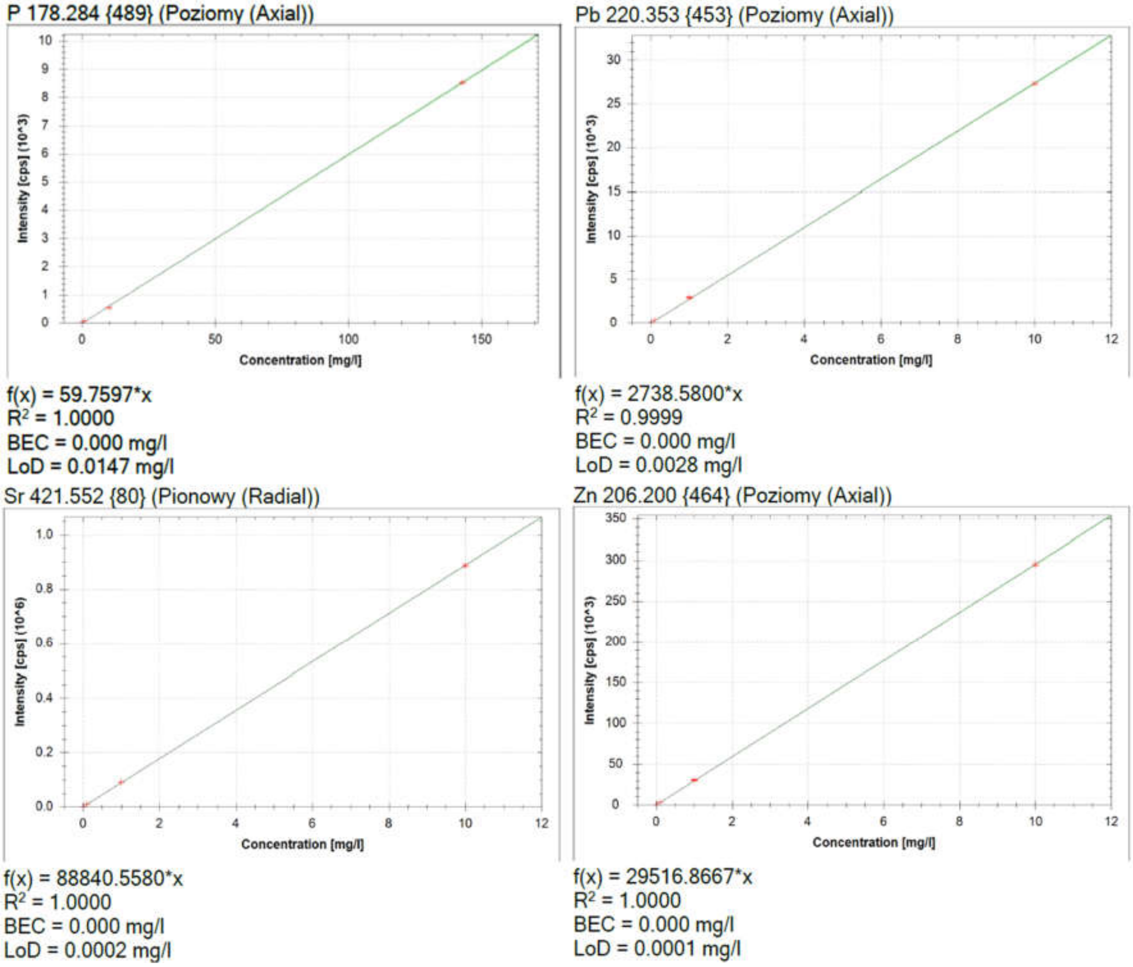

Figure S1. A standard curve and limit of detection (LoD) for Ca, Cu, Fe, K, Mg, Na, P, Pb, Sr, and Zn by inductively coupled plasma atomic emission spectroscopy (ICP AES) on ICAP 7400, Thermo Scientific
